# Applying Diagnostic Stewardship to Proactively Optimize the Management of Urinary Tract Infections

**DOI:** 10.3390/antibiotics11030308

**Published:** 2022-02-24

**Authors:** Faiza Morado, Darren W. Wong

**Affiliations:** 1 Department of Pharmacy, Keck Medical Center, University of Southern California (USC), Los Angeles, CA 90033, USA; faiza.morado@med.usc.edu; 2 Division of Infectious Diseases, Keck School of Medicine, University of Southern California (USC), Los Angeles, CA 90033, USA

**Keywords:** diagnostic stewardship, antimicrobial stewardship, urinary tract infection

## Abstract

A urinary tract infection is amongst the most common bacterial infections in the community and hospital setting and accounts for an estimated 1.6 to 2.14 billion in national healthcare expenditure. Despite its financial impact, the diagnosis is challenging with urine cultures and antibiotics often inappropriately ordered for non-specific symptoms or asymptomatic bacteriuria. In an attempt to limit unnecessary laboratory testing and antibiotic overutilization, several diagnostic stewardship initiatives have been described in the literature. We conducted a systematic review with a focus on the application of molecular and microbiological diagnostics, clinical decision support, and implementation of diagnostic stewardship initiatives for urinary tract infections. The most successful strategies utilized a bundled, multidisciplinary, and multimodal approach involving nursing and physician education and feedback, indication requirements for urine culture orders, reflex urine culture programs, cascade reporting, and urinary antibiograms. Implementation of antibiotic stewardship initiatives across the various phases of laboratory testing (i.e., pre-analytic, analytic, post-analytic) can effectively decrease the rate of inappropriate ordering of urine cultures and antibiotic prescribing in patients with clinically ambiguous symptoms that are unlikely to be a urinary tract infection.

## 1. Introduction

Antibiotics have been arguably the most significant advancement in medicine within the past century with an impact spanning all aspects of medicine allowing for major advances in both surgical and intensive care. However, the widescale utilization of antibiotics has come with the problem of overutilization where an estimated 20–50% of all antibiotics prescribed in hospitals within the United States (US) are deemed either inappropriate or unnecessary [1]. Inappropriate antibiotic use has a risk of multiple complications including adverse drug reactions, *Clostridium difficile* infections (CDI), and a crisis of antibiotic-resistant infections. The Centers for Disease Control and Prevention (CDC) has estimated that 2.8 million antibiotic resistant infections occur annually resulting in more than 35,000 deaths [2]. Antibiotic resistance is, unsurprisingly, driven by long durations of broad-spectrum antibiotic exposure resulting in an increased likelihood of developing antibiotic resistance with prolonged microbiome and morbidity implications [2,3].

Antibiotic stewardship programs (ASP) have become the cornerstone in reconciling the priority of optimizing antibiotics to achieve the best clinical outcome for patients while reducing the development of antibiotic resistance and adverse drug effects [4]. Additionally, antibiotic stewardship initiatives have a key role in maintaining the viability of our current antibiotic armamentarium. Consequently, ample literature has shown the effectiveness of ASP in the reduction of institutional antibiotic resistance rates, reductions in CDI, and significant reductions in healthcare costs [5,6,7,8,9,10,11,12]. These successes resulted in the 2016 endorsement by the Centers of Medicare and Medicaid Services (CMS) requiring development and implementation of ASP in all hospitals, followed by The Joint Commission (TJC) issuance of a new Medication Management Standard in 2017 with a goal of expanding ASP to all hospitals, nursing care centers, and eventually ambulatory care centers [13,14].

The current ASP model has centered upon prospective audit of selected broad-spectrum antibiotics in a pre-prescription model and post-prescription case-by-case feedback, educational initiatives, and evidence-based practice guidelines [15]. ASP processes are resource intensive and require dedicated effort from a collaborative care team including infectious disease physicians, clinical pharmacists, microbiologists, infection control and epidemiologists, and healthcare administration. Additionally, the review of a case of potential inappropriate antibiotic use is triggered after drug administration in a post-prescription manner. This presents a significant challenge due to high workload necessity for daily screening of institutional antibiotic utilization. Moreover, post-prescription review can present difficulties in reaching consensus with clinical care teams on antibiotic discontinuation when clinical ambiguity is often present and possesses an innate delay in awaiting microbiologic data. Despite the impact of ASP, antibiotic resistance continues to increase globally [2,16]. Therefore, while new antibiotic drug development is a key necessity, novel therapeutics cannot keep up with the demand, thereby making the integration of new technologies to empower antibiotic stewardship programs a promising and high yield opportunity [16].

Diagnostic stewardship (DS) is a novel concept focusing on coordinated interventions with a particular attention on the integration of laboratory and molecular diagnostics at an earlier point in care to facilitate improved patient management [17]. Consequently, the downstream effect of earlier interventions includes a reduction in antibiotic usage and subsequently reduced antibiotic resistance. This highlights the symbiotic relationship between DS, antimicrobial stewardship, and infection control initiatives.

Laboratory testing is the single highest volume medical activity and is noted to contribute to the largest growing segment of the US healthcare budget [18,19]. However, an estimated one-fifth of laboratory tests are over utilized, unnecessarily adding on to healthcare costs with either no benefit or deleterious effect to patient care [20]. DS initiatives can be applied to all three phases of laboratory testing: the pre-analytic, at the time of ordering a laboratory test; the analytic, performance of a laboratory test; and the post-analytic stage, the interpretation, and reporting of laboratory results [18,19]. In this way, DS serves the dual purpose of improvement in patient care while also resulting in a cost-effective reduction in healthcare costs [21].

Irrespective of the laboratory testing phase, DS initiatives are intended to take place before an antibiotic is ordered. As a result, this maximizes the impact in days-of-therapy reduction compared to prospective audit and feedback strategies. The majority of DS literature describes the implementation in bloodstream infections; however, a challenging and under-reported application of DS is in the diagnosis and management of urinary tract infections (UTIs).

UTI is amongst the most common bacterial infections in the community and hospital setting, accounting for nearly 10 million healthcare visits and 100,000 hospitalizations per year [22,23,24]. Consequently, the cumulative healthcare cost for the diagnosis and management of UTIs is estimated to be between 1.6 and 2.14 billion annually in the US alone [25]. However, despite its impact on healthcare expenditures, diagnosis of UTIs remain a challenge. Poorly defined clinical diagnostic criteria, an overreliance on laboratory results when lacking clinical symptomatology, and poor consensus on recommended treatment are significant contributors to the variation in antibiotics prescribed and frequency of unnecessary or unnecessarily prolonged antibiotic treatment [26].

Urine cultures are frequently ordered in response to non-specific symptoms and often lack a valid clinical indication [22,26]. Previous studies have identified that a major contributor to unnecessary urine collection is a knowledge gap regarding appropriate triggers for urine testing [27,28]. Indeed one study found that in patients discharged from a pediatric emergency room with antibiotics and a presumptive diagnosis of urinary tract infection, only 51% had resultant urine diagnostics positive for both pyuria and a positive urine culture [29]. This is concordant with another observational emergency department study where clinical and microbiologic evidence of a urinary infection was lacking in almost 70% of patients discharged with a presumptive diagnosis of urinary tract infection [30]. A prospective audit conducted at two academic medical centers revealed that 68% of urine cultures were ordered without a clear clinical indication. Urine cultures were obtained due to non-specific findings such as confusion (12%), leukocytosis (21%), history of a UTI (11%), foul smelling or discolored urine (9%), urinary retention (8%), weakness or dizziness (7%), and dysglycemia (4%) [31]. Secondly, it can be difficult to differentiate UTIs from asymptomatic bacteriuria (ASB). Compounding the problem is that ASB is a common finding with incidence reported from 5–15% of healthy asymptomatic adults and reaching up to 50% in elderly residents of long-term care facilities [32]. Additionally, in patients with indwelling urinary catheters, the incidence of ASB increases at the rate of 2 to 7% per day and approaches nearly 100% in patients with chronic catheterization [32]. This rate is further compounded by the frequency of contaminated urinary samples. In 1998, a survey of 906 institutions reported urine culture contamination rates reaching 36%. A follow-up survey in 2008 showed no significant progress had occurred in the reduction of contamination rates, and in fact, the rate of contaminated specimens in low-performance laboratories had increased to an average of 42% [24]. The misinterpretation of urine analyses and urine culture results lead to inappropriate prescribing and overexposure of antibiotics with one systemic meta-analysis estimating treatment of ASB occurring in 45% of cases [33].

Critically, the treatment of ASB has not been shown to improve patient outcomes nor does it decrease either the frequency of symptomatic UTI or prevent future occurrences of ASB [31]. Therefore, current guidelines recommend against the routine screening for ASB and against treatment except in special circumstances [32]. Highlighting this finding, due to the high incidence of ASB in elderly residents of long-term care facilities, one study found that routine submission of urinary cultures was almost arbitrary and urine culture and treatment did not have any appreciable functional improvement in resident activities of daily living (ADL) score [34]. However, despite these recommendations, frequent overtreatment of catheter- and non-catheter-associated ASB continue to occur with increases particularly notable in the ambulatory and telemedicine setting [35,36,37]. Concurrently, unnecessarily prolonged duration of antibiotics are often prescribed, occurring in over 60% of cases in some series [37]. This ambiguity of symptoms, the commonality of positive urine cultures, and prolonged duration of antibiotic prescriptions all combine to make a UTI a prime target for DS intervention with a high potential impact in patient care and cumulative antibiotic reduction. 

## 2. Methods

We conducted a systematic review of the literature with a focus on the application of molecular and microbiological diagnostics, clinical decision support, and implementation of antibiotic stewardship initiatives for urinary tract infection. A search was performed in PubMed including search terms ‘diagnostic stewardship’ AND ‘antibiotics’ AND ‘urinary tract infection.’ References from identified articles were also reviewed to secondarily identify other relevant studies.

## 3. Results

**Figure 1:**
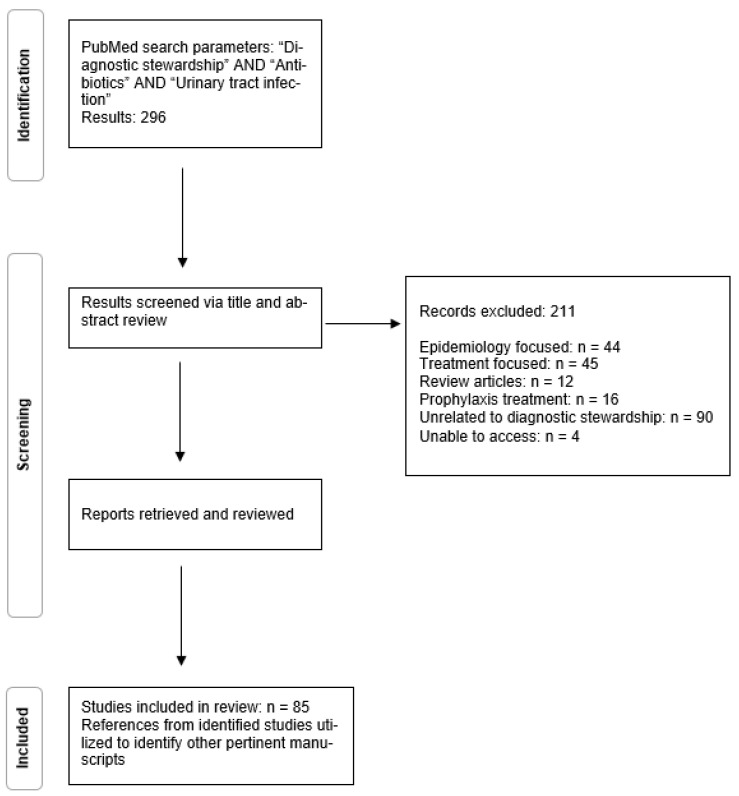
Literature Review Methodology.

A total of 296 results were identified following initial PubMed search. The literature search was performed 9/26/21 and restricted to articles in the English language. These results were reviewed for relevance with specific attention to articles focusing on the implementation of diagnostic stewardship methods as they applied to a urinary infection. Identified articles were screened for relevance. Following review, 211 results were excluded with indication described in Figure 1. The remaining 85 results were obtained and reviewed in depth with references from identified articles a source of secondary relevant results. Applications of diagnostic stewardship were divided into pre-analytic, analytic, and post-analytic interventions.

### 3.1. Pre-Analytical Urinary Stewardship Interventions

Pre-analytical interventions center around the principle of limiting inappropriate ordering of urinalyses and urine cultures. This priority directly eliminates the risk of false-positive culture results due to sampling error, specimen contamination, or misinterpretation of laboratory result. Urine cultures are commonly ordered as a ‘pan-culture’ strategy for non-specific or ambiguous clinical symptoms. As a result, several institutions have begun to utilize computerized physician order entry (CPOE) and clinical decision support (CDS) alerts as a means of operationalizing DS tactics to combat this behavioral practice. In a quasi-experimental study, Keller and colleagues implemented a best practice alert recommending against urine testing in asymptomatic individuals and against the treatment of ASB [38]. The alert was automatically generated for laboratory orders of urinalyses or urine cultures and for antibiotics commonly ordered for a UTI. While this intervention required minimal resources, the bundled intervention resulted in a statistically significant reduction in urine cultures (18.2% pre-intervention vs. 11.8% post-intervention) and antibiotic orders (4.4% pre-intervention vs. 3.9% post-intervention) [38]. Lee and colleagues expanded on this idea and required providers to indicate if their patient was considered as part of the designated high-risk population (i.e., pregnancy) requiring treatment for ASB as recommended by the Infectious Diseases Society of America (IDSA). Patient samples meeting pre-specified criteria underwent a standard culture process. If patients did not meet eligibility criteria or clinicians opted not to specify the patient category, patient samples were subjected to screening in the analytical phase for pyuria or bacteriuria before culture. Overall, this stepwise intervention was independently associated with a reduction in antimicrobial use (adjusted odds ratio 0.76, *p* < 0.001) [39]. In contrast, Demonchy et al. described the use of an emergency department computerized decisional support program which was triggered by a listed diagnosis of urinary tract infection. However, while improved adherence to practice guidelines occurred, the benefit was limited and 41% of physicians bypassed and thereby did not use the program [40].

Leveraging CPOE for DS efforts have also proven beneficial for patients with indwelling urinary catheters. In a large academic medical center, a guideline regarding appropriate urine culture indications in catheterized patients was created and made readily accessible for reference in the electronic medical record (EMR) [41]. Appropriate indications for urine culture included suprapubic pain or tenderness, acute gross hematuria, costovertebral angle tenderness, increased spasticity or autonomic dysreflexia in patients with altered neurologic sensation, new fever or rigors without an identified alternative infectious foci, or acute mental status change with clinical examination negative for an alternative, more likely etiology. Requests for urine culture required clinician input of an indication, which resulted in a 34%, statistically significant, reduction in urine cultures post-intervention [41]. Additionally, Page et al. reported a novel approach in a 33-bed neurocritical care unit where initial orders for urine culture were screened by the charge nurse prior to specimen collection. In patients meeting appropriate institutionally determined criteria, urine cultures were sent. In patients who did not meet the criteria, the critical care clinician was called to review the necessity of urine culture. During the 30-day pilot program, 76% of urine cultures were reviewed with the attending clinician. Of this proportion, 57% were deemed not to be required with a consequent statistically significant (70%, *p* = 0.037) reduction in catheter-associated urinary tract infection (CAUTI) rate without any identified increases in adverse effects [42].

While implementation of CPOE and CDS tools appear to result in a modest reduction in unnecessary urine culture requests, studies have shown that DS pre-analytic strategies were most effective when coupled with antibiotic stewardship-led educational support and clinician/infectious disease physician direction in devising appropriate EMR order prompts [20]. Effective interventions include a lecture series with case-based vignettes, institutional developed pocket cards with easy-to-reference diagnostic algorithms for UTI, and evidence-based guidelines with preferred treatment regimen and duration of therapy [22,25,35,43,44,45,46,47]. One example of the effectiveness of educational objectives involved a multi-interventional approach. A lecture to the department of surgery and department of medicine on appropriate urine culture diagnostics was held, followed by individualized educational monthly sessions with nursing leadership from each care unit, information-technology (IT) computer screensaver reminders, and random chart review by infection control staff with monthly provider-specific feedback. This was combined with hospital administration support and ultimately resulted in a near 50% reduction in urine culture orders (post-interventional 280 compared to 509 urine cultures monthly) without any observed change in patient mortality [44]. Similarly, Trautner et al. implemented a streamlined diagnostic algorithm for ASB and CAUTI combined with case-based audit and feedback at a Veterans Affairs (VA) health system. This resulted in a significant reduction in urine cultures compared to a contemporaneous control group at another VA hospital (41.2 compared to 23.3 per 1000 bed days) [48]. Even more significant was that this reduction was sustained after the completion of the intensive implementation period when direct audit and prospective clinician feedback was replaced with a quarterly educational case presentation [48]. Choi et al. described a similar quasi-experimental study of a pharmacist-led audit and feedback approach improving appropriateness of outpatient antibiotic prescription in skin and urinary tract infections [49]. Educational sessions have also been shown to be effective with statistically significant decreases in treatment of asymptomatic bacteriuria in long-term care facilities and nursing homes [50,51]. This underscores the practicality of implementation of stewardship initiatives in outpatient care settings.

In addition to ensuring appropriate clinical indications for culture, the reduction in contaminant cultures is another high-yield DS target. A College of American Pathologists survey of 127 laboratories, which defined contaminated urine specimens as having 2 isolates in quantities greater than 10,000 CFU/mL, found contamination rates varied significantly with an average contamination rate of 42% in the 10^th^ percentile, 1% in the 90th percentile, and 15% at the median institution [52]. A significant variation did exist, but post-collection refrigeration of the specimen had a substantial effect in the rate of contamination with an average reduction of 50% [24,52]. Educational initiatives on proper urine specimen collection were an important finding in laboratories with lower rates of contamination. Additionally, in urine specimens stored at room temperature for 4 h there was an increase in culture colony counts by 10%, but for those in storage for 24 h this rate increased by over 135% [53]. In patients with delayed processing, three observational studies found 96–100% specificity concordance with negative culture in specimens held for 24 h preserved with boric acid compared to immediate culture [53]. As an example, one healthcare system identified an average urine culture contamination rate of 28%. A bundled intervention including standardization of specimen collection with education of staff and patients, expedited transport to the lab, implementation of preservatives, and a standardized definition and tracking mechanism for urine culture contamination resulted in a reduction in contaminant rates below a target rate of 5% [24].

### 3.2. Analytical Urinary Stewardship Interventions

An estimated 70 to 80% of urinary samples intended for culture yield negative results for the diagnosis of a UTI [54]. Therefore, several health systems have adopted a reflex urine culture (RUC) approach to reduce the cost and resources associated with unnecessary urine culture testing and treatment of ASB. RUC refers to the process of limiting urine cultures to situations in which the urinalysis meets pre-specified criteria.

RUC has been shown to reduce rates of urine cultures, ASB, and antimicrobial use with no increase in Gram-negative blood stream infections in quasi-experimental and single center studies [55,56,57,58,59,60,61]. One benefit of RUC programs is that it has been implemented and studied in a variety of settings including the emergency department, hospitals (including intensive care units), long-term care, and ambulatory clinics. However, while it may be flexible in use, the optimal urinalysis cut-off for RUC remains undefined. In surveys conducted among acute care and community hospitals, half of the respondents indicated that their health systems actively use RUC methods. However, pre-specified criteria to designate an abnormal urinalysis warranting a reflex culture varied significantly for each institution [62,63]. A significant challenge in implementing RUC is the lack of evidence-based guidelines defining optimal urinalysis parameters that are predictive of positive urine cultures. In the absence of uniform recommendations, several studies have evaluated the role of various urinalysis parameters as a DS tool for reflex cancellation of urine culture.

In particular, the absence of pyuria on urinalysis has been boasted as an excellent diagnostic aid in excluding UTI from a differential with a negative predictive value (NPV) consistently reported above 90% [56,59,64]. As a result, RUC protocols tend to include pyuria as a trigger for reflex cultures. However, the minimum white blood cell count per high-power field (WBC/HPF) to define pyuria has widely varied in available studies. The most used cutoffs were >5 WBC/HPF and >10 WBC/HPF. While some studies suggest higher pyuria cut-offs of >25WBC/HPF or >50 WBC/HPF may result in a larger decrease in the rate of urine cultures performed, such cut-offs are not well-studied [55,57].

In an outpatient setting with a 9.5% (262/2764) prevalence of bacteriuria, a positive urinalysis defined as nitrite positive and/or with the presence of ≥5 WBC/HPF and bacteria, exhibited a strong association with a positive urine culture and resulted in a NPV of 91% [59]. In clinical settings with higher reported prevalence of bacteriuria, the NPV remained above 90%. Fok and colleagues aimed to determine the predictive leukocyte count for a positive urine culture in 874 male urology outpatients in which 20% were known to have a positive urine culture. Pyuria defined as >5 WBC/HPF was present in 163/176 (93%) positive samples and yielded a NPV of 97%. Utilizing this definition of pyuria would have avoided 69% of unnecessary cultures while missing only 7% of positive urine cultures [64]. Such a high NPV suggests that a urinalysis negative for pyuria, will likely not result in a positive urine culture in settings with ≤20% prevalence of bacteriuria [65]. However, the 7% risk of false-negative testing found in the study by Fok et al. may not be a clinically acceptable miss rate for some clinicians. Lowering the risk of false-negative results may be accomplished by incorporating other urinalysis parameters such as the presence of nitrite, leukocyte esterase, or bacteria [65,66]. Jones and colleagues aimed to develop and implement a UA screening algorithm highly predictive for urine culture positivity in 1546 patients aged 5 years and older presenting to the emergency department (ED) in which the prevalence of bacteriuria was reported to be 20%. In a univariate analysis, the presence of >10 WBC/HPF, positive nitrites, positive leukocyte esterase, or positive bacteria were highly predictive of a positive urine culture (*p* < 0.001) [56]. When used individually, the NPV of each UA parameter was 92, 86, 93 and 96%, respectively. Alternatively, when based on the presence of one or more of these criteria, the NPV increased to 98.2% and would result in a reflex culture cancellation in 604/1546 (39%) samples, with only 11 false-negative results of 314 positive cultures (3.5%) [56].

When deciding upon a definition for a positive urinalysis, hospitals will have to decide on whether they will implement a more permissive or restrictive definition based on comfortability with false-negative rates, but also potential for stewardship impact. In a quasi-experimental study conducted at six different acute care hospitals within the VA network, rates of urine cultures performed were stratified for permissive versus restrictive RUC policies [55]. One site utilizing a permissive definition of a positive urinalysis allowed culturing if a sample met any of the following criteria: positive leukocyte esterase, positive nitrite, or >5 WBC/HPF. The sites using a restrictive policy allowed urine culturing only when urinary samples contained >10 WBC/HPF, without considerations for the presence leukocyte esterase or nitrite. Overall, a RUC program was associated with a 21% decrease in the rate of urine cultures performed (*p* ≤ 0.01). However, when stratified, a larger decrease in the rate of urine cultures was observed for sites with more restrictive criteria compared to permissive. Post-intervention monthly average rates of urine culture changed from 27.1 to 13.8 per 1000 patient days (*p* < 0.01) in sites using a restrictive definition compared to a change from 57.2 to 48.8 per 1000 patients days in more permissive sites (*p* ≤ 0.05) [55]. Similarly, in three hospitals, the implementation of a reflex urine culture strategy requiring ≥10 WBC/HPF resulted in a decrease in monthly urine cultures by 47% [67]. Other considerations when formalizing a conditional urine culture policy is the assessment of specimen quality. In a survey conducted across community hospitals, three institutions reported using epithelial cells as criteria for a poor-quality specimen to be rejected [63]. The presence of epithelial cells may indicate that the sample was incorrectly collected and contaminated with skin flora which may result in an inappropriate reflex to urine culture [63].

In order to support DS efforts, Munigala and colleagues successfully developed new, more restrictive definitions of reflex urine culture tests and integrated them into the EMR to decrease unnecessary testing. The new reflex tests were emailed to providers and imbedded into commonly used order sets (i.e., admission to medical intensive care unit). Pre-intervention, a urinalysis would trigger a reflex culture if it contained the presence of protein, blood, nitrite, or leukocyte-esterase. Post-intervention, urinalysis parameters of proteinuria or hematuria were excluded as they are not typically associated with UTIs, and instead a urinalysis would reflex to culture only if the sample was positive for either nitrite or leukocyte esterase. Leveraging the power of the EMR resulted in a 45% reduction in urine cultures performed post-intervention [61].

While not as extensively studied, literature suggests that urinalysis is an acceptable method to effectively exclude a UTI as an infectious cause and reduce the rate of inappropriate urine culturing in patients with indwelling urinary catheters [57,61,65,68,69,70]. In the study carried out by Munigala and colleagues, there was an overall 45% reduction in urine cultures, but the effect was most marked for catheterized (75.6%) specimens compared to clean catch specimens (37.8%). Additionally, a urine reflex culturing protocol implemented across three VA medical centers would have reflexively cancelled 29% of urine cultures reducing the CAUTI rate from 1.82 infections per 1000 catheter days to 1.30 infections per 1000 catheter days [57]. Another large tertiary care hospital reported that a reflex urine culture protocol implemented in five ICU settings significantly decreased monthly urine culture (*p* < 0.001) and CAUTI rates (*p* = 0.04) for 12 months post-intervention [68].

While RUC can be a useful diagnostic aid of exclusion, it should be emphasized that the benefit of using a UA to rule out an infection is in the context of the patient exhibiting signs and symptoms consistent with a UTI. The key to improving overall diagnosis and management of a UTI is assessing and identifying signs and symptoms associated with a UTI prior to urine testing. Additionally, while a negative urinalysis indicated by the absence of pyuria may have a high NPV for infection, a positive urinalysis does not accurately indicate the presence of an infection. Pyuria alone is not a reliable predictor of a UTI as it simply indicates the presence of inflammation within the urinary tract [32]. Only in its absence can it exclude UTI, particularly if pre-test probability is low. 

It is also important to recognize that while a negative urinalysis can be a reliable predictor of a negative urine culture in most patient populations, a false-negative rate of 4 to 9% may not be clinically acceptable in patient populations where urinary tract colonization may pose harm [56,59,64]. Therefore, some health systems have elected to exclude the following individuals from RUC policies: pregnant, immunocompromised, and undergoing urinary tract instrumentation [64,68]. Additionally, if the pre-test probability of a UTI is high, clinicians can elect to override the protocol and proceed with urine culture testing [68].

Several studies have also sought to integrate systemic biomarkers into a urinary tract infection diagnostic algorithm. However, due to poor sensitivity and poor predictive utility they have either been found, as with C-reactive protein, to have no utility [71] or with procalcitonin, a limited role, in potentially shortening the duration of antibiotic exposure in patients with febrile disease syndromes due to a urinary infection source [72,73]. Several alternative biomarkers including adenosine-5′-triphosphate, urinary xanthine oxidase, and urinary myeloperoxidase have been investigated as possible predictors for levels of urinary bacteria. However, none have shown sufficient reproducible sensitivity and specificity to be recommended [74,75].

An important and increasingly powerful analytic tool are rapid diagnostic platforms. Flow cytometry provides an automated method to calculate the number of bacteria and leukocytes in urine samples. However, distinguishing between bacteriuria and infection remains a challenge, with variability arising from differences in specimen collection and prior antibiotic use contributing to false-positives due to flow cytometry measurement of dead bacteria [74]. Mass spectrometry, MALDI-TOF (Matrix-assisted laser desorption ionization-time of flight mass spectrometry) is also a powerful tool utilized to identify bacteria directly from urine specimens without the need for culture incubation. Burillo et al. reported a major error rate of 4% with MALDI-TOF compared to positive urine culture [76]. Efforts to improve upon the limitation of both technologies has resulted in attempts to combine both flow cytometry and MALDI-TOF. Wang et al. described a strategy using flow cytometry to identify urine samples with bacterial counts greater than ≥10^5^ CFU/mL upon which MALDI-TOF was utilized to identify bacterial species. When utilized on 1456 urine specimens, this strategy was 94% consistent with conventional culture [77]. Similarly, Zboromyrska et al. used flow cytometry to identify appropriate samples for MALDI-TOF which subsequently correctly identified 86% of cases. Identified specimens via MALDI-TOF had disk diffusion sensitivity testing performed resulting in results one day sooner than conventional methods [78].

### 3.3. Post-Analytical Urinary Stewardship Interventions

Limiting prescriber ordering of urine cultures to patients with a high probability of a UTI can be onerous, requiring a complete reconceptualization of long-standing practices such as pan-culturing as part of a “fever work-up.” Inciting such behavioral change can be complex, challenging, and resource intensive, and therefore it may take time for effective behavioral change to be realized.

An alternative method aimed at immediately decreasing antimicrobial usage after an inappropriate culture is ordered, thus bypassing physician ordering practices, is selective reporting of microbiology laboratory results. The Clinical and Laboratory Standards Institute (CLSI) describes selective susceptibility reporting, also known as cascade reporting, as a strategy of suppressing secondary agents on antimicrobial susceptibility test (AST) results unless the organism is resistant to primary, narrow-spectrum agents [79,80]. This approach is supported by the IDSA and Society for Healthcare Epidemiology of America (SHEA) as a less resource intensive means to direct clinicians to use the least toxic and most narrow-spectrum agent available [80]. The purpose of selective reporting of AST results is to preserve the clinical viability of broad-spectrum agents when narrow-spectrum options are susceptible and to improve the appropriateness of antibiotic prescriptions. To investigate the impact of laboratory reporting on antibiotic prescription, 113,780 urine cultures from 48 laboratories were reviewed to determine the association of susceptibility reporting and antibiotic prescribing in the empirical (1–3 days prior to culture result) and directed window (0–5 days after culture result). Despite variation in laboratory reporting practices, reporting a specific antibiotic’s susceptibility had an increased odds ratio of prescribing that antibiotic in both the empirical (adjusted odds ratio, 1.23) and directed window (adjusted odds ratio, 2.98) [81]. This impact in empiric treatment decisions provides support for “nudging” behaviors to preferred optimal antibiotic selection [82].

The addition of selective susceptibility reporting to laboratory testing policies has been shown to favorably modify prescribing decisions on a patient level [83,84,85,86,87]. In a randomized control case-vignette study, medical residents were provided either a full-length or an abridged, selective reporting of AST results. Due to selective reporting, the appropriateness of antibiotic prescriptions increased from 7 to 41% depending on the clinical scenario [84]. Using actual antimicrobial usage data, McNulty and colleagues demonstrated a 20% prescribing increase in cephalexin (OR1.20, 95% CI 1.12–1.30) and an 8% decrease in amoxicillin-clavulanic acid (OR 0.92, 95% CI 0.89–0.96) after modifying AST results to report cephalexin in place of amoxicillin-clavulanic acid [84]. Additionally, selective reporting of AST results can minimize the selection of drug-resistant strains by avoiding broad-spectrum agents when the isolate is susceptible to narrower-spectrum antibiotics. In a study carried out by Langford et al., ciprofloxacin susceptibility was suppressed for all *Enterobacteriaceae* from all sources if the organism was susceptible to other agents on the Gram-negative panel [87]. In a secondary analysis evaluating *Escherichia coli* susceptibility to ciprofloxacin, selective reporting was associated with an immediate and sustained reduction in ciprofloxacin usage with mean monthly utilization decreasing from 87 defined daily doses (DDD) per 1000 patient days at baseline to 39 DDD per 1000 patient days post-implementation of selective reporting. Reduction in ciprofloxacin use coincided with statistically significant improvement in *E. coli* susceptibility to ciprofloxacin. Notably, 80% of *E. coli* isolates were from urinary tract sources and were likely a key factor for observed changes in utilization and susceptibility [87].

While selective reporting can guide clinicians in choosing more narrow-spectrum agents, it does not address the unnecessary treatment of ASB as clinicians are more likely to prescribe antibiotics for a positive culture if antibiotic susceptibilities are available [84]. Leis et al. conducted a proof-of-concept study in which positive urine culture results from low-risk, non-catheterized patients were not automatically reported. Rather, the culture report was modified to read “The majority of positive urine cultures from inpatients without an indwelling urinary catheter represent [ASB]. If you strongly suspect that your patient has developed a urinary tract infection, please call the microbiology lab” [85]. The initial suppression of culture and susceptibility results decreased clinician predilection to treat positive culture results while allowing them to pursue results in patients with a high pre-test probability of a UTI. Following the modified reporting intervention, treatment of ASB decreased from 48 (95% CI 32–65%) to 12% (95% CI 5–57%) for an absolute risk reduction of 36% (95% CI 15–57%, *p* = 0.002) [85]. Daley et al. introduced a similar modified report in which clinicians could obtain urine culture results by directly contacting the microbiology laboratory. The proportion of appropriately treated UTIs was higher in the intervention group compared to the control group: 80.0% versus 52.7%, respectively (absolute difference = −27.3%; RR 0.42; *p* = 0.002) [88]. While suppressing all urine culture results can improve UTI prescribing practices, such methods can burden microbiology labs with an overwhelming number of requests.

In addition to selective reporting of AST results, IDSA emphasizes the use of local susceptibility data to guide prescriber decisions for empiric antimicrobial choices [81]. Previous studies have shown that syndrome or site specific antibiograms (i.e., urine) lead to improved prescribing patterns for empiric therapy [89]. One institution published a local urinary antibiogram to highlight recommended empiric treatment options for a UTI and created a new order set and clinical decision support system based on collated antibiogram data. Prior to implementation, fluoroquinolones displayed an unfavorable susceptibility profile but accounted for 45% (IQR 41.0–49.0) of all antibiotic prescriptions for outpatient treatment of UTIs monthly. A reduction in fluoroquinolone use was observed post-intervention and encompassed only 32.0% (IQR 28.9–35.1) of monthly UTI antibiotic prescriptions (*p* < 0.001). Concomitantly, monthly beta-lactam prescriptions increased from 14.0% (IQR 11.5–16.5%) to 24.5% (IQR 21.6–27.4%) post-intervention (*p* < 0.001), resulting in greater concordance with the local antibiogram [90].

### 3.4. Diagnostic Strategies for Urinary Tract Infections in Development

With a 95% sensitivity and 85% specificity at growth thresholds as low as 10^2^ CFU/mL, the standard urine culture with sensitivity has been the clinician gold standard for diagnosing UTIs [91]. Although a clinically validated diagnostic tool, standard urine culture with sensitivity is not without its limitations. The current process takes 48 to 72 h to receive organism identification with associated susceptibility data, allowing for a longer than necessary course of a potentially ineffective or overly broad agent [22,91,92]. Additionally, there has been controversy over the growth threshold to define a clinically significant and positive urine culture. It is debated that the traditional threshold of > 10^5^ CFU/mL is too high and will result in a missed UTI diagnosis as 25 to 40% of women with symptoms consistent with a UTI will present with a negative urine culture at this threshold [91,92]. Moreover, while standard urine culture techniques can reliably identify *E. coli*, they struggle to detect other uropathogens such as *Enterococci* and *Group B streptococci* with positive predictive values of 10 and 33%, respectively [92]. Furthermore, traditional culturing methods are highly prone to contamination with periurethral, vaginal, and perineal flora during collection, with institutions across the US reporting an average contamination rate of at least 15% [22,91,92].

New technologies are being developed to help address inadequacies of standard urine culturing methods. Multiplex polymerase chain reaction (mPCR) is a highly appealing technology given its high sensitivity and specificity and fast turn-around-time of results. These techniques detect bacteria via PCR amplification and subsequent identification of conserved 16s ribosomal genes. Several companies are evaluating the role of PCR directly on urine samples allowing for rapid UTI diagnostics. Altobelli et al. utilized a biosensor array with a limited panel of probes to detect 16s rRNA bacterial sequences on urinary clinical specimens. As a proof of concept, the negative predictive value was 99% [93]. One pilot study assessed the feasibility of using a commercially available qualitative PCR blood pathogen test to identify urinary pathogens. The PCR assay resulted 43 h prior to culture results with a sensitivity and specificity of 82 and 60%, respectively [94,95]. While clinically appealing, PCR-based technologies would add onto existing laboratory costs of urine cultures as a compliment to traditional methods. Although PCRs can provide faster organism identification, urine cultures are still required to confirm antibiotic sensitivities. 

An alternative diagnostic method under investigation is next-generation sequencing (NGS), which matches microbial DNA to large curated species libraries of a particular microbiome, such as the urinary microbiome [91]. Benefits of NGS is that it can provide the bacterial load of each organism present in the sample as well as their associated antibiotic resistance genes. This can help guide therapy by considering the predominant pathogenic organism as compared to a qualitative PCR assay [92]. However, similar to a PCR, NGS does not provide comprehensive antibiotic sensitivity data and often requires standard culture-based antibiotic sensitivity testing for phenotypic confirmation.

The proper integration of molecular diagnostic technologies has not been defined, particularly regarding urinary tract infection. Additionally, molecular testing lacks the ability to differentiate between dysbiosis, bacteriuria, and infection. Therefore, standardized criteria to interpret NGS and PCR results will need to be established.

The advent of rapid diagnostics has given AS programs another platform to optimize patient care and further improve efficiency of AS initiatives. As antimicrobial drug development slows down and concerns for antibiotic resistance continues to increase, AS programs are forced to improve antibiotic usage through innovative approaches such as the implementation of new molecular technologies such as PCR and NGS. 

## 4. Discussion: Recommended Evidence-Based Strategies for the Implementation of Diagnostic Stewardship in Urinary Infections

**Figure 2:**
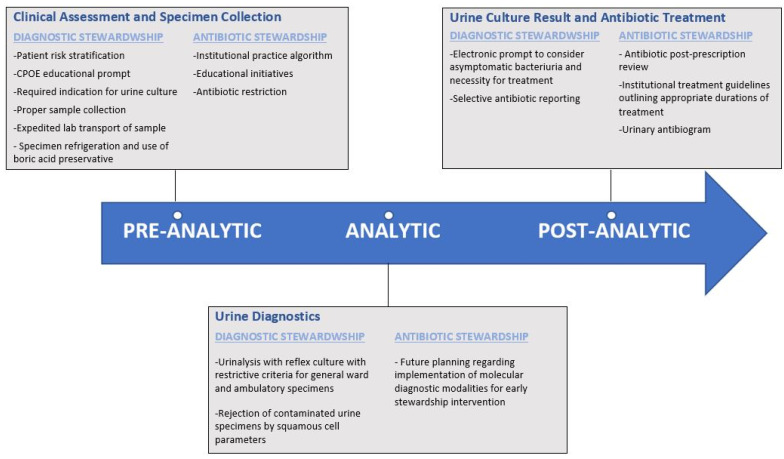
Recommended strategy to integrate and align diagnostic stewardship and antibiotic stewardship interventions for urinary tract infection.

Antibiotic stewardship programs are now widely implemented in hospitals in accordance with CMS requirements. However, ASP has had a more limited impact for suspected urinary infection due to the lack of consensus diagnostic criteria and the prevalence of positive urine cultures. Optimizing antibiotics for a urinary infection and reducing the reliance on overly broad-spectrum antimicrobial regimens continues to be a challenging, unmet need. Implementing diagnostic stewardship to existing antibiotic stewardship programs seeks to meet this need with active interventions to align antibiotic use with appropriate necessity and selection. Critically, diagnostic stewardship can be applied to the inpatient, urgent care, and ambulatory setting. This is particularly meaningful for skilled nursing facilities, long-term care, and ambulatory care that are much more limited with respect to dedicated prospective antibiotic stewardship review. Ultimately, diagnostic stewardship initiatives require institutional and administration support along with collaboration between physicians, pharmacists, microbiology lab personnel, and information technology staff. Based on the available literature, we propose evidence-based high-yield interventions (Figure 2) that have shown evidence of benefits in patient care, but also, a cost-effective reduction in healthcare expenditures.

The first target should achieve a goal of ensuring urine cultures are obtained for appropriate indications. This method eliminates the largest source of inappropriate antibiotic use in response to a coincidentally positive urine culture in an otherwise asymptomatic patient. We recommend the use of dedicated educational sessions focused on guiding physicians away from routine urine culture for scenarios where they are of low utility such as routine pre-operative urinalysis prior to orthopedic or non-urologic surgeries, dialysis patients with oliguria or anuria, or clearly asymptomatic patients. While all educational objectives have shown some benefit, it appears that case-based vignette series involving active learning were the most common, more effective, and the most sustainable modality. Additionally, current literature has shown a significant reduction in urine cultures via modification of the CPOE system. We recommend a best practice alert of the current evidence-based indications for urine culture at the time of ordering. This would be followed by a requirement for the clinician to input which of these indications the patient meets for culture. These interventions should be supplemented by clinician feedback, potentially with selected audit of different inpatient wards in a monthly rotating manner. This approach would provide education on best practices, reinforce these objectives at the time of urine culture order, and provide focused feedback on performance to clinicians. The approach by Page and colleagues, with initial urine culture order subjected to charge nurse review prior to specimen collection appears highly promising; however, this may not be applicable on a large scale. Therefore, a modified approach would involve tracking urine culture volume by care unit which would provide an opportunity for targeted implementation of this strategy in units that have excess numbers of inappropriate urine cultures or high incidence of inappropriate treatment. Integrating this approach with antibiotic stewardship measures, specifically an institutional recommended treatment regimen based on the current antibiogram and expected practice duration of therapy, would further optimize these interventions. Similarly, we would encourage education of patients and nursing staff on appropriate urine specimen collection highlighting the cleansing of the periurethral and perineal area prior to obtaining a urine specimen as well as the strict avoidance of specimens obtained from urine catheter bags. Streamlining microbiology laboratory workflow via expedited specimen transport, specimen refrigeration at 2–8 °C, and in the cases of delayed processing, the utilization of a boric acid preservative may further reduce the potential for specimen contamination.

The second approach would be to implement a cascade of utilizing an initial urinalysis with cultures only performed if pyuria is present. Direct urine culture orders would only be recommended for pregnant patients or those with clearly defined neutropenia. While there are merits in both a restrictive versus permissive culture policy, if amenable it may be prudent to utilize a permissive culture algorithm in intensive care patients, and a more restrictive criteria in general medical wards or ambulatory patients. Additionally, we find it reasonable to reject urine specimens that have clearly elevated squamous cells indicative of a contaminated sample.

Finally, selective antibiotic cascade reporting should be an easily implementable opportunity. Preferred narrow-spectrum agents should be reported with a preference for first-line recommended options such as nitrofurantoin, trimethoprim-sulfamethoxazole (TMP/SMX), and fosfomycin. Result reporting should be in congruence with institutional best-expected practice treatment regimens. While requiring a phone call to the microbiology lab to obtain culture results did result in a significant reduction in antibiotic prescription, this approach did not seem feasible for widespread applicability. However, including a reminder in the electronic urine culture report about the possibility of asymptomatic bacteriuria would be a simple opportunity to reduce potential antibiotic administration in patients with low suspicion of a UTI. 

At this point, rapid diagnostics have been limited by cost and a lack of consensus criteria in how to interpret results. The sequential flow cytometry and MALDI-TOF appear to be a promising method to combine both technologies. Molecular diagnostics and molecular resistance testing have been more readily adapted to bloodstream infection; this technology has not been adapted to urinary infections. Applying molecular diagnostics to urine infections will be a significant challenge due to the prevalence of positive cultures and often polymicrobial cultures as opposed to blood specimens which should in general be sterile. Therefore, it would seem plausible that NGS and PCR technology would need to be paired with initial screening tests (i.e., reflex testing after identification of pyuria) to maximize the pre-test probability for infection. Molecular testing on urine, particularly with resistance gene testing, may allow for the de-escalation of broad spectrum or carbapenem therapy in alignment with antibiotic stewardship interventions. However, maximizing the power of molecular diagnostics will be a challenge due to the increasing short duration (as short as 3 days) of therapy that is recommended for the treatment of routine cystitis. Additionally, molecular diagnostics are unlikely to provide sensitivities for preferred cystitis treatments (i.e., nitrofurantoin), and urine culture will still be required.

Ultimately, a UTI is the most common bacterial infection in the community and hospital setting and is a prime source of unnecessary laboratory testing and antibiotic overutilization. Antimicrobial stewardship initiatives are resource intensive and limited by the inherent challenge of the ambiguity of symptoms prompting urine cultures and the significant volume of testing. Therefore, adopting a bundled, multidisciplinary, and multimodal diagnostic stewardship approach spanning the three phases of laboratory testing can augment antibiotic stewardship and result in a meaningful reduction in healthcare costs with improved patient outcomes. 

## Data Availability

The data presented in this study are available on request from the corresponding author.
